# BCI-Based Rehabilitation on the Stroke in Sequela Stage

**DOI:** 10.1155/2020/8882764

**Published:** 2020-12-13

**Authors:** Yangyang Miao, Shugeng Chen, Xinru Zhang, Jing Jin, Ren Xu, Ian Daly, Jie Jia, Xingyu Wang, Andrzej Cichocki, Tzyy-Ping Jung

**Affiliations:** ^1^Key Laboratory of Advanced Control and Optimization for Chemical Processes, Ministry of Education, East China University of Science and Technology, Shanghai, China; ^2^Department of Rehabilitation, Huashan Hospital, Fudan University, Shanghai, China; ^3^Guger Technologies OG, Austria; ^4^Brain-Computer Interfaces and Neural Engineering Laboratory, School of Computer Science and Electronic Engineering, University of Essex, Colchester, Essex CO4 3SQ, UK; ^5^Skolkowo Institute of Science and Technology (SKOLTECH), 143026 Moscow, Russia; ^6^Systems Research Institute PAS, Warsaw, Poland; ^7^Nicolaus Copernicus University (UMK), Torun, Poland; ^8^Institute for Neural Computation and Institute of Engineering in Medicine, University of California San Diego, La Jolla, CA, USA

## Abstract

**Background:**

Stroke is the leading cause of serious and long-term disability worldwide. Survivors may recover some motor functions after rehabilitation therapy. However, many stroke patients missed the best time period for recovery and entered into the sequela stage of chronic stroke.

**Method:**

Studies have shown that motor imagery- (MI-) based brain-computer interface (BCI) has a positive effect on poststroke rehabilitation. This study used both virtual limbs and functional electrical stimulation (FES) as feedback to provide patients with a closed-loop sensorimotor integration for motor rehabilitation. An MI-based BCI system acquired, analyzed, and classified motor attempts from electroencephalogram (EEG) signals. The FES system would be activated if the BCI detected that the user was imagining wrist dorsiflexion on the instructed side of the body. Sixteen stroke patients in the sequela stage were randomly assigned to a BCI group and a control group. All of them participated in rehabilitation training for four weeks and were assessed by the Fugl-Meyer Assessment (FMA) of motor function.

**Results:**

The average improvement score of the BCI group was 3.5, which was higher than that of the control group (0.9). The active EEG patterns of the four patients in the BCI group whose FMA scores increased gradually became centralized and shifted to sensorimotor areas and premotor areas throughout the study.

**Conclusions:**

Study results showed evidence that patients in the BCI group achieved larger functional improvements than those in the control group and that the BCI-FES system is effective in restoring motor function to upper extremities in stroke patients. This study provides a more autonomous approach than traditional treatments used in stroke rehabilitation.

## 1. Introduction

Stroke is one of the most common cerebrovascular diseases worldwide. It causes numerous problems and is the leading cause of serious and long-term disability in many countries [1]. Motor disorders, including hemiplegia of the upper limbs, are a frequent consequence of stroke. Therefore, timely and effective treatments are needed for functional motor recovery to help survivors perform daily activities better. However, many stroke patients missed the best time period for recovery and entered the chronic sequela stage. Effective treatments for patients after stroke are a challenge.

Different approaches have been used for poststroke rehabilitation, including conventional therapy, robotic therapy, stem cell therapy, noninvasive brain stimulation techniques, and other novel therapies [2, 3]. However, during conventional rehabilitation, there is no objective way to determine whether the patients are performing the expected motor imagery task [4]. Although the rehabilitation may result in an improvement in upper limb mobility and function, this benefit did not persist months after stroke [2].

Brain-computer interface (BCI) can directly translate brain activity to specific commands and are reaching their technological maturity [5]. It could provide an alternative communication way to disabled patients. For instance, reference [6] proposed a peripheral-display speller based on BCI technology for amyotrophic lateral sclerosis (ALS) patients, which yielded a performance comparable to the conventional matrix-based speller. In addition, motor imagery- (MI-) based BCI, one of the widely used BCI systems, could also be used in stroke rehabilitation to translate brain signals into intended movements [7]. Different from other paradigms such as event-related potentials (ERP) [8, 9] and steady-state visual evoked potentials (SSVEP) [10], MI by the BCI user elicits an event-related (de)synchronization (ERD/S) in the electroencephalogram (EEG), which represents the result of conscious access to the content of the intention of a movement [11–13]. Therefore, the ERD/S features may be used to detect motor intention in stroke patients. Additionally, the strength and topology of the ERD/S have been shown to vary as a function of the degree of upper limb motor impairment in stroke patients [14], suggesting that it may also be used as an objective correlate of stroke recovery.

MI, the mental rehearsal of physical movement tasks, represents a new approach to access the motor system and rehabilitation at all stages of stroke recovery [15, 16]. Some groups have tested the applicability of MI-based BCI in stroke rehabilitation and demonstrated clinical improvements [17, 18]. Different from the preceding results, a magnetoencephalography- (MEG-) based BCI system was used for stroke rehabilitation and six out of eight stroke patients could control the system, but no significant motor improvements were found [19]. Moreover, to help patients modulate their brain activity proficiently, many training methods with feedback have been developed to attempt to improve the performance of MI-based BCIs [4, 20]. The feedback provided by these systems typically includes playing rewarding sounds or a real-time visual display and may not directly or accurately reflect the true intention of the patient. To provide patients with closed-loop sensorimotor integration for motor rehabilitation, functional electrical stimulation (FES) has been explored as a feedback stimulus in some studies [20, 21]. In order to induce muscle movement to improve or restore muscle function, FES uses pulses of electrical current to stimulate one or more muscle groups and can provide efficient proprioceptive feedback [21]. In reference [4], a rehabilitation system using FES as a feedback stimulus was investigated. The results show that EEG activity in the patients' motor cortex was significantly increased. In reference [20], researchers used an FES system and a bar as the feedback. After 10 training sessions, one stroke patient partially regained control of dorsiflexion in her paretic wrist.

In many studies, patients with shorter onset time were selected since it is hard to recover for patients in the sequela stage. In addition, single pathway feedback is the main form of feedback used in existing research. There is some evidence that viewing real or artificial body parts results in a stronger desynchronization than viewing nonbody part movements [22, 23]. Hence, we selected stroke patients in the sequela stage and sought to investigate the benefits of BCI for stroke rehabilitation using a multimodal feedback paradigm, in which cues were presented to patients via audio stimuli, by virtual limbs, and via FES.

## 2. Material and Methods

### 2.1. BCI-FES System

**Figure 1:**
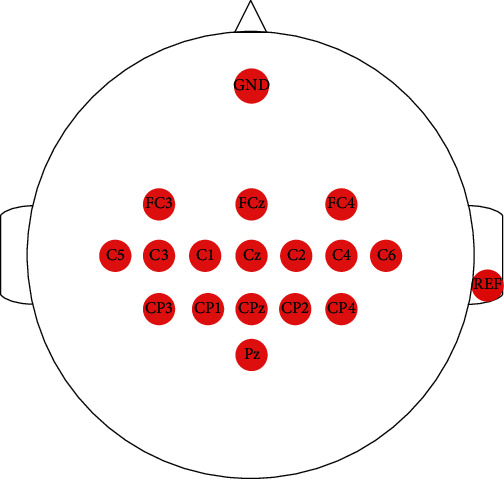
The electrode distribution used in this study.

This study used the recoveriX system (g.tec medical engineering GmbH, Austria) for experiments, which is a BCI-FES system. In the BCI system, 16 channels (FC3, FCz, FC4, C5, C3, C1, Cz, C2, C4, C6, CP3, CP1, CPz, CP2, CP4, and Pz) were used for EEG signal recording, as shown in Figure 1. The ground electrode (GND) was placed over the forehead (FPz), and the reference electrode (REF) was located at the right earlobe. The signals were sampled at 256 Hz through a g.USBamp (g.tec medical engineering GmbH, Austria). The band-pass filter was set to 0.1-30 Hz. It stimulated appointed muscle groups, with the stimulation modes triggered by the BCI system.

**Figure 2:**
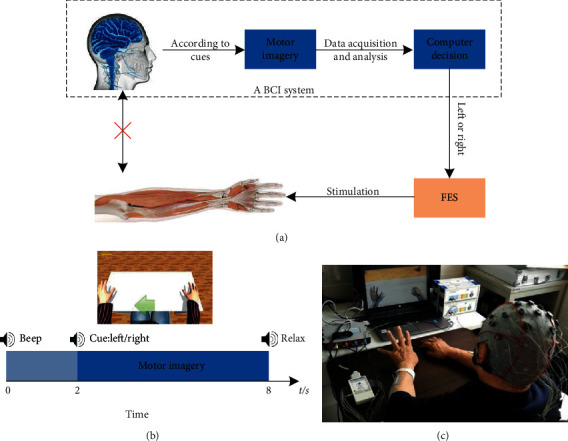
(a) The schematic of the BCI-FES system. (b) The timing of a trial of the motor imagery paradigm. Each trial consisted of task and rest periods. A patient started to execute motor imagery tasks upon the appearance of the cue (“left” or “right”). A virtual avatar of one patient's upper limbs was used to provide virtual reality feedback. (c) This picture shows the scene of BCI-FES rehabilitation training for one patient.

Figure 2(a) shows the schematic of the BCI-FES system. As stroke patients suffer neurological damage, the brain regions associated with motor functions might be compromised and unable to control limb movements directly. The BCI system attempts to acquire, analyze, and recognize the EEG data of motor imagery from patients. The FES system will then be activated if the BCI system detects that the user is imagining hand movement on the instructed side of the body. The muscle contraction caused by FES is calibrated to be sufficient to cause movement in the affected hand. In this study, the FES muscle stimulators were placed in the extensor carpi radialis longus, extensor carpi radialis brevis, and extensor carpi ulnaris to improve the wrist dorsiflexion function of patients [24]. Before each run, stimulation parameters (current amplitude and pulse duration) were calibrated as follows. First, we set the pulse duration of the current to 300 *μ*s. Then, we increased slowly the current amplitude until the stimulators could visibly cause wrist dorsiflexion. Verbal feedback was obtained from the patients about the strength of muscle stimulation in order to prevent the excessive current from the fatigue of wrist muscles.

### 2.2. Participants

Sixteen stroke patients in the sequela stage were randomly assigned to the BCI group (*n* = 8) and the control group (*n* = 8). The two groups received routine rehabilitation training, including limb dominance exercise, muscle tension, and limb control training, three times a week, for four weeks. On this basis, the BCI group underwent motor imagery rehabilitation training using the BCI-FES system. All participants signed a written consent form prior to this experiment. The local ethics committee approved the consent form and experimental procedure before any patient participated. All participants were right-handed, and they were diagnosed by computerized tomography (CT) or magnetic resonance imagining (MRI), without cognitive disorder and any unsuitable diseases for receiving the BCI-FES system.  Table 1 shows the demographic information for all participants (P1-P8 were in the BCI group and P9-P16 were in the control group).

### 2.3. Experimental Procedure

The participants sat in a comfortable chair in a shielded room after being prepared for EEG recording. During data acquisition, participants were asked to relax and avoid unnecessary movements. Instructions were delivered through both sounds and visual cues. The experimenter informed the participants that they would hear cues over a speaker that would instruct them to imagine either their left or their right wrist dorsiflexion. At the same time, the screen showed the hand avatar, which reflected the participants' movements to help them imagine. As shown in Figure 2(b), each trial lasts eight seconds and starts with a warning “beep.” Two seconds later, the cue (the command to imagine a left or right wrist dorsiflexion) was played to ask participants to perform kinesthetic motor imagery. Six seconds later, a “relax” command was presented, informing participants that the trial was over. During the imagining time, the FES would be activated if the BCI system detected the user was imagining movement on the instructed side. The muscle contraction initiated by the FES was sufficient to cause dorsiflexion on the affected wrist. The feedback period lasted four seconds, and the intertrial interval lasted two seconds.

Figure 2(c) shows the scene of BCI-FES rehabilitation training for a sample patient. When the BCI detected the patient had performed the correct MI task upon the appearance of the cue (“left” or “right”), the avatar would give him/her visual feedback and the FES would be activated to cause the wrist dorsiflexion of the corresponding side. Three rehabilitation sessions were carried out in a week (spaced evenly across the week). Each session contained two runs. Each patient participated in sixty trials within one recording run. Before each session, the participants needed to answer two questions: (1) Did you have a good rest last night? (2) Do you feel well now? After each session, the participants needed to answer two questions: (1) Are there any problems during this session? (2) How did you feel after this session?

### 2.4. Pattern Recognition

For motor imagery feature extraction, the EEG data were filtered using a fifth-order Butterworth band-pass filter from 8 to 30 Hz, since this frequency band included the range of frequencies that were mainly involved in performing motor imagery [22, 25].

We used the common spatial pattern (CSP) algorithm as a feature extraction method. CSP has been widely used in processing EEG data from motor imagery [26–30]. Consider the EEG signal *E*_*i*,*c*_ ∈ *ℝ*^*N*×*S*^ of the *i*-th trial in class *c*, where *N* and *S*, respectively, represent the number of channels and the number of sampling points. The spatial covariance matrix of the class *c* is computed as follows:

(1) $$\begin{array}{c} \Sigma_{c}=\frac{1}{n_{c}}\overset{n_{c}}{\underset{i=1}{\sum }}\frac{E_{i,c}E_{i,c}^{T}}{\text{trace}\left(E_{i,c}E_{i,c}^{T}\right)}, \end{array}$$

where *n*_*c*_ represents the number of trials in class *c*.

CSP is based on the simultaneous diagonalization of two covariance matrices. It finds a spatial filter *w* to maximize variance for one class and minimize variance for the other class at the same time:

(2) $$\begin{array}{c} \underset{w}{\max}J\left(w\right)=\frac{w^{T}\Sigma_{1}w}{w^{T}\Sigma_{2}w}\text{s}.\text{t}.{\left\|w\right\|}_{2}=1. \end{array}$$

Using the Lagrange multiplier method, Eq. (2) is transformed into the generalized eigenvalue problem:

(3) $$\begin{array}{c} \Sigma_{1}w=\lambda \Sigma_{2}w. \end{array}$$

where *λ* and *w* are the generalized eigenvalue and eigenvector, respectively.

The spatial filters of CSP *W* ∈ *ℝ*^*N*×2*m*^ are formed by eigenvectors which are corresponding to *m* maximum and minimum eigenvalues. This study used the first three and last three columns (eigenvectors) of the projection matrix as the spatial filter to compute the features. The EEG data of the single trial *E* can be transformed into:

(4) $$\begin{array}{c} Z=W^{T}E. \end{array}$$

The feature *f*_*p*_ can be obtained from *Z*_*p*_ (*p* = 1, ⋯, 2*m*):

(5) $$\begin{array}{c} f_{p}=\log\left(\frac{\mathrm{var}\left(Z_{p}\right)}{\sum_{i=1}^{2m}\mathrm{var}\left(Z_{i}\right)}\right). \end{array}$$

In the classification scheme, we applied linear discriminant analysis (LDA) as the classifier. It finds a linear combination of features that characterizes or separates two classes [31–34]. Discriminant scores are calculated by a discriminant function:

(6) $$\begin{array}{c} L_{p}=\arg\underset{L_{p}}{\max}\frac{{L_{p}}^{T}S_{B}L_{p}}{{L_{p}}^{T}S_{W}L_{p}}, \end{array}$$

where *S*_*B*_ and *S*_*W*_ are, respectively, the between-class and within-class scatter matrices.

(7) $$\begin{array}{c} S_{B}=\overset{2}{\underset{i=1}{\sum }}N_{i}\left(m_{i}-m\right){\left(m_{i}-m\right)}^{T},\\ S_{W}=\overset{2}{\underset{i=1}{\sum }}\underset{f_{p}\in C_{i}}{\sum }\left(f_{p}-m_{i}\right){\left(f_{p}-m_{i}\right)}^{T}, \end{array}$$

where *N*_*i*_ is the number of the samples in class *C*_*i*_ (*i* = 1, 2), *N* is the number of all samples, *m*_*i*_ = (1/*N*_*i*_)∑_*f*_*p*_∈*C*_*i*__*f*_*p*_ is the mean of the samples in class *C*_*i*_, and *m* = (1/*N*)∑_*f*_*p*__*f*_*p*_ is the mean of all the samples. This study used the data in the first run to train the LDA classifier, and then, the classifier could be used in a subsequent online run.

### 2.5. Functional Assessment

This study used the Fugl-Meyer Assessment (FMA) to evaluate the motor function of the upper limb control in the participants. This scale is an index to assess the sensorimotor impairment in individuals who have had a stroke. It was first proposed by Axel Fugl-Meyer and his colleagues as a standardized assessment test for poststroke recovery [35]. It has been tested several times and is found to have excellent consistency, responsivity, and good accuracy [36–38]. It is now widely used for clinical assessment of motor function. The FMA assesses several impairment dimensions using a 3-point ordinal scale (0 = cannot perform; 1 = can perform partially; 2 = can perform fully).

## 3. Results

### 3.1. Classification Performance Comparison

**Figure 3:**
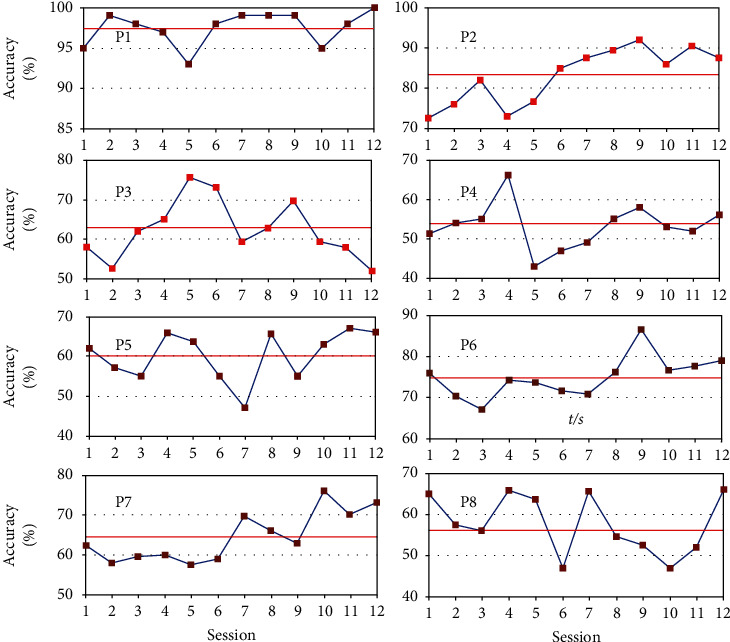
The accuracies across 12 sessions for all participants in the BCI group. The red line indicates the average accuracy.

Figure 3 presents the BCI classification performance (as measured by accuracy) across 12 training sessions for the eight participants in the BCI group. Results showed that most of the participants could get better performance in the last session than in the first session. The average motor imagery accuracy of the eight participants in the last session was 72.9%, an improvement of 5.0% from the first session.

Specifically, for participants P1, P2, and P7, the average accuracies in the last session were, respectively, 100%, 88.3%, and 73.3%; their performances were visibly improved from the first session (95%, 73.3%, and 61.7%). P1 achieved the best and most stable performance of all participants. An interesting observation is that, before motor imagery-based rehabilitation training, P1 had usually imagined the hand movements according to his report. In fact, P1 is clinically diagnosed with minor depression and often imagines his body parts. Hence, he is good at concentrating on motor imagery tasks and achieved the best performance of all participants.

Interestingly, the performance of participants P3, P4, P5, and P8 showed relatively large fluctuations. In particular, the performance of participant P3 in the last session was worse than in the first session. This may be because the young participant P3 felt the training boring after too many repeated sessions, and thus, she was not very motivated and dedicated in the later part of the training. For other participants, the undulating accuracies may be related to emotional fluctuations or normal intersession variability (nonstationarity) in EEG signals or noise causes. Hence, the average accuracies of them were lower than others in the two categories of motor imagery.

### 3.2. Functional Improvement

Table 2 shows the FMA scores before and after rehabilitation training over the two groups. Four participants' FMA scores have increased in the BCI group (the proportion is 50%), while three participants in the control group also exhibited increases in FMA (the proportion is 37.5%). Before rehabilitation training, the average score in the BCI group (19.5) was relatively lower than that in the control group (20.6). After 12 training sessions, the average score of the BCI group (23.0) was significantly higher than that of the control group (21.5). On the whole, the average score in the BCI group has been increased by 3.5, while the average score in the control group has been only raised by 0.9. The results also show that the BCI-FES rehabilitation training was significantly effective.

In terms of individual performance, the scores for participants P1, P6, P7, and P8 in the BCI group were observed to increase throughout the experiments; the scores for participants P10, P11, and P15 in the control group were also observed to increase. The scores for the remainder of the participants did not change. In particular, for participants P6 and P7 in the BCI group, the period after stroke onset was over 2 years. The health condition of these participants should have tended to be stable and difficult to improve using routine rehabilitation training [39]. However, after the BCI-FES rehabilitation training, certain motor functions were restored and the scores were also improved for them. Among the participants in the BCI group, P4 achieved the lowest score (8) after rehabilitation training, which is consistent with the former result that he got the worst average accuracy.

Paired one-tailed *t*-tests were used to show the differences between FMA scores before and after the rehabilitation for the two groups, respectively. After four weeks of rehabilitation training, the scores in the BCI group have been increased (mean = 3.5, *p* = 0.049), while the scores in the control group have been increased a little (mean = 0.9, *p* = 0.044).

In summary, improvements in motor functions have been achieved for some of the participants. The participants in the BCI group obtained more improvements than those in the control group (3.5 vs. 0.9).

### 3.3. EEG Patterns

This study used power spectral densities and topographic maps extracted by CSP to detect motor imagery EEG patterns.

**Figure 4:**
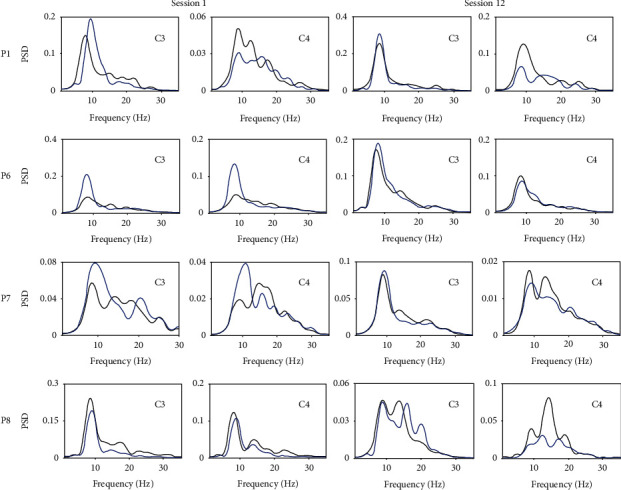
The power spectral density maps from electrodes C3 and C4 for four participants in the BCI group (blue: right motor imagery, black: left motor imagery).

Figure 4 shows the power spectral density (PSD) maps from electrodes C3 and C4 for the four participants in the BCI group, whose FMA scores were increased throughout the experiments. These two electrodes have been shown to record important characteristics of motor imagery [40, 41]. The PSDs in session 1 and session 12 were averaged over multiple trials (60 left-wrist dorsiflexion trials and 60 right-wrist dorsiflexion trials).

For participant P1, the PSD maps were consistent with the theory presented by Pfurtscheller and colleagues [11, 42] in both session 1 and session 12. At electrode C3, the EEG energy during right motor imagery was higher than the EEG energy during the left motor imagery, while at electrode C4, the situation was the opposite. For participants P6, P7, and P8, there were no significant differences in the EEG energy at electrodes C3 and C4 in session 1. It was thus difficult to distinguish the two motor imagery tasks. In session 12 of BCI-FES rehabilitation training, the EEG energy for these participants became similar to that observed for participant P1.

**Figure 5:**
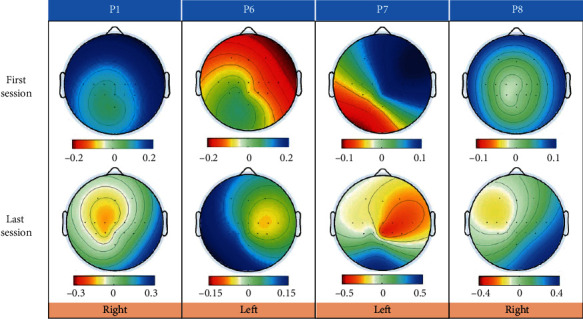
Topographic maps from four participants illustrating the first and last spatial patterns extracted by the CSP method.

Figure 5 showed the topographic maps from the paretic side of the previously mentioned participants illustrating the first and last spatial patterns extracted by the CSP method (P1 and P8: right motor imagery; P6 and P7: left motor imagery). In CSP, *W* is the projection matrix, and *W*^−1^ is the inverse matrix of *W*. The columns of *W*^−1^ are the time-invariant vectors of EEG source distribution vectors called common spatial patterns. The first pattern was obtained by maximizing the variance of the right motor imagery, which was associated with the ERD phenomenon over the left sensorimotor area of the cortex. Accordingly, the ERD phenomenon over the right motor area was associated with the last pattern, corresponding to the left motor imagery.

The results for participant P1 showed a clear ERD phenomenon in the left cerebral cortex during the right motor imagery. For participant P6, larger regions were initially active in session 1: the ERD phenomenon almost occupied the whole contralateral hemisphere. However, the ERD phenomenon became more centralized and was mainly distributed around electrode C4 in the last session, and the ERS phenomenon was observed in the left cerebral cortex. The results for participant P7 did not show a clear pattern from the contralateral hemisphere for left motor imagery in the first session. However, the map of the last session showed a clear ERD phenomenon in the right cerebral cortex. For participant P8, in both the first and last session, the ERD phenomenon was not particularly strong and occurred to the left of the central area for the first session and the upper left cerebral cortex for the last session.

By tracking the changes in the motor imagery EEG patterns during rehabilitation, we tried to explore cortical reorganization. Study results suggested that after rehabilitation, the sensorimotor cortex in the contralateral hemisphere could be activated. Figure 5 also indicated that areas around electrodes C3 and C4 were strongly associated with the left- and right-hand motor imageries, which was consistent with the neurophysiology phenomenon reported in [25, 41].

## 4. Discussion

In the experimental procedures of many studies, an arrow pointing left or right was used as the cue. However, some evidence suggests that viewing real or artificial body parts results in a stronger desynchronization in the EEG during attempted movement/motor imagery [22, 23]. Hence, this study used both virtual limbs and FES as feedback, which, we hypothesize, could help participants perform the motor imagery tasks better.

In data analysis, researchers have used the functional Magnetic Resonance Imaging (fMRI) approach to assess brain function after BCI therapy [43–46]. This study, instead, used EEG signals. Unfortunately, we have to note that not all participants were able to gain appreciable improvements in classification performance. Such results are in line with the literature [47]. Besides, classification performance did not exactly correspond with FMA scores. For example, although the average classification performance was poor (<60%) for participant P8, his FMA score was increased by 5. The reasons that half of the participants in the BCI group had not made any progress may be as follows: (1) Participants felt fidgety and bored after too many repeated sessions of the rehabilitation training and could not concentrate on motor imagery (for P4 and P5). (2) The original score was so high that it was difficult to improve (for P3). (3) Another possible reason could be the small sample sizes used and the short training time. Adding more participants or increasing the training time was difficult. On the one hand, most participants are reluctant to use systems that are not yet widely applied. On the other hand, if the training time becomes longer, it is hard for participants to stick with it.

This study also explored the changes in the EEG patterns as an index to objectively assess the efficacy of the BCI-FES rehabilitation training. After 12 sessions of the training, PSD maps for four of the participants became sufficiently distinct to allow our BCI classifier to differentiate the two motor imagery tasks; the active patterns in the topographic maps gradually became more centralized and shifted to the sensorimotor areas (around channels C3 and C4) and the premotor areas (around channels FC3 and FC4). Some literature has reported a similar phenomenon. For example, Tam et al. reported that some stroke patients were not able to produce focal ERD patterns in sensorimotor areas and that the active patterns were produced in frontal premotor areas and parietal areas [48]. This observation reveals the rehabilitation mechanism: functionality initially occurs in larger regions but gradually returns to the motor or nearby cortical regions during recovery. This could be a sign of cortical reorganization or neuroplasticity in the affected hemisphere.

Several limitations of our study merit further discussion. First, as to EEG signal analysis, this study may ignore the effect of nonstroke factors on the classification performance, such as fatigue [49–52] during rehabilitation. In future work, we seek to improve the experimental design to enhance the enthusiasm of the participants. For instance, we can design some simple games like the literature [53] and also can give the participants additional bonus based on their success rate in the experiment. Second, this study was unable to explain why four of the eight participants in the BCI group did not improve. The same situation also appeared in the literature [54], and none of the previous studies was able to explain the exceptions. Third, the number of training sessions (12) is low. However, we could not increase the session number due to the limited hospital stay duration of the participants. Last but not least, the FES muscle stimulators were only placed to trigger wrist dorsiflexion. They also could be placed in other muscle groups in order to help motor functional rehabilitation of other body parts such as the elbow, knee, and ankle. Their rehabilitation effect is still unknown, pending further research.

## 5. Conclusion

This study combined a motor imagery-based BCI and an FES system to provide stroke patients with closed-loop sensorimotor integration for motor rehabilitation. Both virtual limbs and FES were used as feedback, which could help patients improve their training through visual and sensory pathways. Our results showed that participants in the BCI group obtained more improvements than the participants in the control group. This study provides a more autonomous approach than traditional treatments for stroke rehabilitation. Additional research is needed to enhance the portability of the BCI-FES system.

## Figures and Tables

**Table 1:** Demographic information about the participants.

Group	ID	Gender	Age	Stroke type	Paretic side	Stroke onset (months)
BCI	P1	Male	58	Hemorrhage	Right	6
BCI	P2	Female	70	Ischemic	Right	8
BCI	P3	Female	22	Trauma	Left	24
BCI	P4	Male	65	Ischemic	Right	8
BCI	P5	Male	44	Hemorrhage	Right	22
BCI	P6	Male	45	Hemorrhage	Left	24
BCI	P7	Male	30	Trauma	Left	38
BCI	P8	Male	56	Ischemic	Right	16
Control	P9	Male	52	Infarction	Left	14
Control	P10	Male	64	Infarction	Left	6
Control	P11	Male	65	Infarction	Right	13
Control	P12	Female	54	Infarction	Right	9
Control	P13	Female	25	Trauma	Right	20
Control	P14	Male	72	Hemorrhage	Left	14
Control	P15	Male	40	Hemorrhage	Right	6
Control	P16	Male	30	Infarction	Left	7

**Table 2:** FMA score comparison before and after rehabilitation training over the two groups.

BCI	Pre-FMA	Post-FMA	Stroke onset (months)	Control	Pre-FMA	Post-FMA	Stroke onset (months)
P1	24	27	6	P9	20	20	14
P2	10	10	8	P10	30	32	6
P3	39	39	24	P11	21	24	13
P4	8	8	8	P12	14	14	9
P5	14	14	22	P13	36	36	20
P6	20	35	24	P14	4	4	14
P7	24	28	38	P15	18	20	6
P8	17	23	16	P16	22	22	7
AVG	19.5 ± 9.9	23.0 ± 11.4	18.3 ± 10.9	AVG	20.6 ± 9.7	21.5 ± 10.0	11.1 ± 5.0

## Data Availability

The (EEG) data used to support the findings of this study are available from the corresponding author upon request.
